# Comparing the clinical performance and cost efficacy of [^68^Ga]Ga-PSMA-11 and [^18^F]PSMA-1007 in the diagnosis of recurrent prostate cancer: a Markov chain decision analysis

**DOI:** 10.1007/s00259-021-05620-9

**Published:** 2021-11-13

**Authors:** Ian Alberts, Clemens Mingels, Helle D. Zacho, Sabine Lanz, Heiko Schöder, Axel Rominger, Marcel Zwahlen, Ali Afshar-Oromieh

**Affiliations:** 1 Department of Nuclear Medicine, Inselspital, Bern University Hospital, University of Bern, CH-3010 Bern, Switzerland; 2 Institute of Social and Preventive Medicine, University of Bern, Bern, Switzerland; 3 Department of Nuclear Medicine and Clinical Cancer Research Center, Aalborg University Hospital, Hobrovej 18-22, DK-9000 Aalborg, Denmark; 4 Molecular Imaging and Therapy Service, Memorial Sloan Kettering Cancer Center, New York, NY USA

**Keywords:** Markov chain analysis, PET/CT, Positron emission tomography, PSMA, Recurrent prostate cancer

## Abstract

**Purpose:**

Amongst others, [^68^Ga]Ga-PSMA-11 and [^18^F]PSMA-1007 are available for the detection of recurrent prostate cancer (rPC). There are currently limited data comparing the performance of these two radioligands with respect to clinical outcomes or their cost efficacy, which this study aims to address.

**Methods:**

Two hundred and forty-four patients undergoing PSMA PET/CT for rPC were retrospectively analysed for this study (one hundred and twenty two with each radiopharmaceutical) to generate rates of PET positivity, negativity and unclear findings. Patients underwent follow-up to determine the rate of additional examinations and to confirm PET findings. A Markov chain decision analysis was implemented to model clinical decision-making processes and to analyse clinical performance of the two tracers. We determine their clinical cost efficacies using cost data from several countries where both radiotracers are in routine use.

**Results:**

The PET positivity rate was non-significantly higher for [^18^F]PSMA-1007 compared to [^68^Ga]Ga-PSMA-11 (91.8% vs. 86.9%, *p* = 0.68), whereas the rate of uncertain findings was significantly greater (17.2% vs. 8.25%, *p* = 0.02). The probability of a true positive finding was higher for [^68^Ga]Ga-PSMA-11 (0.90, 95% CI 0.70-0.98) vs. [^18^F]PSMA-1007 (0.81, 95% CI 0.66–0.91). A significantly (*p* < 0.0001) higher PPV for [^68^Ga]Ga-PSMA-11 (0.99, 95% CI 0.99–1.0 vs. 0.86) was found compared to [^18^F]PSMA-1007 (0.86, 95% CI 0.82–1.00). Intervention efficacy analysis favoured [^68^Ga]Ga-PSMA-11, where the number needed to image (to achieve a true positive finding) was 10.58 and the number needed to image to harm (to achieve a false positive finding) was − 8.08. A cost efficacy analysis favours [^68^Ga]Ga-PSMA-11 in three of the four jurisdictions analysed where health economic data was available (Switzerland, Israel, Australia) and [^18^F]PSMA-1007 in one jurisdiction (Denmark).

**Conclusion:**

The analysis reveals a non-significantly higher PET positivity rate for [^18^F]PSMA-1007, but finds significantly greater rates of uncertain findings and false positive findings when compared to [^68^Ga]Ga-PSMA-11. We find differences in the two tracers in terms of clinical performance and cost efficacy. The method presented herein is generalisable and can be used with clinical or cost data for other countries or tracers.

**Supplementary Information:**

The online version contains supplementary material available at 10.1007/s00259-021-05620-9.

## Introduction

PET/CT with PSMA radioligands is now firmly established as the preferred modality for the staging of biochemically recurrent prostate cancer (rPC) [1] and is increasingly used for the staging of high-risk primary PC [2]. In addition to the first described [^68^Ga]Ga-PSMA-11 [3], various other PSMA-radioligands have recently become available. The introduction of [^18^F]-labelled ligands was an important development, and these tracers exhibit several advantages when compared to [^68^Ga]Ga-PSMA-11 [4], including increased cyclotron production, lower positron energy and longer half-life when compared to ^68^Ga, theoretically to the benefit of image quality [4]. Furthermore, [^18^F]PSMA-1007 does not undergo significant renal excretion in the first few hours post injection, which is potentially beneficial in the detection of local recurrences, although diuretics in combination with later imaging may also achieve this effect with radioligands undergoing renal excretion [5, 6]. However, given the paucity of comparative imaging data for PSMA ligands, improved patient outcomes when using ^18^F-radioligands remain to be demonstrated [7]. Indeed, some studies have reported some disadvantages, such as a higher rate of indeterminate findings when using [^18^F]PSMA-1007 [8, 9] which could create diagnostic confusion [10]. The relative performance of the various PSMA radioligands with respect to these patient-level outcomes has only been partially investigated [7].

PC is the most commonly diagnosed cancer in men [11] and recurrence post initial treatment is common [12]. Consequently, there is increasing use of and regulatory approval for PSMA-PET/CT in economically developed countries [13] and there is increasing interest in improving access to advanced imaging techniques to improve oncological outcomes in middle-income and developing economies [14, 15]. Given the expense and infrastructure for these resource-intensive imaging modalities, it is incumbent upon the imaging community to deliver clear evidence for efficacy, including the economic rationale for their use. The lack of comparative data with respect to economic or clinical performance of the numerous PSMA radioligands in common use precludes any informed choice and represents an important unmet need in contemporary PC imaging. Whereas a number of analyses have established that imaging with [^68^Ga]Ga-PSMA-11 can be cost-effective compared to conventional imaging [16–18], the claim that ^18^F-radiolabelled radioligands are more cost-effective or exhibit diagnostic superiority in comparison to other tracers has not been subject to systematic testing, despite their widespread implementation. Therefore, the aim of this present study is to compare the clinical performance and cost efficacy of the two PSMA radioligands [^68^Ga]Ga-PSMA-11 and [^18^F]PSMA-1007. We systematically obtain clinical data from patients undergoing PSMA PET/CT with either [^68^Ga]Ga-PSMA-11 or [^18^F]PSMA-1007 to inform a Markov chain decision model. In doing so, we obtain data regarding the relative clinical performance and health-care cost efficacy comparing these two radioligands, as well as providing a generalisable methodology, which can be implemented using clinical or cost data from other countries.

## Materials and methods

The consolidated health economic evaluation reporting standards (CHEERS) statement was followed during the design and execution of this study [19] which was approved by the institutional review board. In this retrospective analysis, we investigated 244 consecutive individuals with biochemically recurrent PC who were referred to our centre for PSMA-PET/CT. Inclusion criteria were individuals referred for the investigation of biochemical recurrence of PC. Exclusion criteria were individuals presenting for primary staging of PC or for assessment for PSMA therapy. Routine PET/CT with [^68^Ga]Ga-PSMA-11 was introduced in Switzerland in January 2017, and [^18^F]PSMA-1007 was introduced at our centre in September 2019 following its temporary approval for use in Switzerland. Cognisant of a learning curve when encountering a new tracer, individuals examined in the first 3 months following the introduction of [^18^F]PSMA-1007 were excluded from the analysis to eliminate this as a source of bias.

**Fig. 1:**
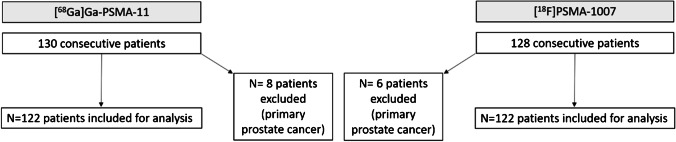
Study flow chart showing patient recruitment, total patients included and excluded to yield to balanced pairs

From January 2020 to May 2020, 130 consecutive individuals undergoing [^18^F]PSMA-1007 were identified, of whom 122 underwent imaging for recurrent PC. Likewise, immediately prior to the change to [^18^F]PSMA-1007 in September 2019, 128 consecutive individuals undergoing [^68^Ga]Ga-PSMA-11 (May 2019–September 2019) were identified, of whom 122 underwent imaging for recurrent PC and included for analysis, thus yielding a matched pair of cohorts. The study flow chart is given in Fig. 1. Details regarding age, Gleason score, initial staging, applied activity and the prostate-specific antigen (PSA) value at time of scanning are as outlined in supplementary Table S1, yielding two matched cohorts of patients with no statistical differences in any characteristic parameters.

### Image routines and evaluation

Imaging procedures are as outlined in the supplementary materials. Clinical imaging reports and all clinical records were scrutinised by the first and second authors. All PET/CT scans were dual reported in consensus by two experienced nuclear medicine physicians. Scans with at least one PSMA-avid lesion suspicious for rPC were recorded as “positive” at a patient-based level, and those with no PSMA-avid lesions suspicious for rPC were recorded as “negative” at a patient-based level (= PET-positivity rate). Clinical notes and minutes of multi-disciplinary team (MDT) meetings were scrutinised to follow-up (min 6 months, max 21 months). Cases where the PET/CT report revealed unclear findings and any further examinations performed were recorded. The clinical notes and radiological information system (RIS) were scrutinised to obtain the results of any additional investigations. Likewise, for “negative” cases, situations where repeat PSMA PET/CT was recommended in the report or requested by the referring physician were recorded.

### Markov chain decision analysis

As shown in Supplementary Figure S1, a connected network of states originating from the patient’s referral to the end state of a diagnosis was outlined, which can be modelled via a time inhomogeneous Markov model [20], which we implemented using Microsoft Excel (details in supplementary materials). Initial modelling assumptions were as follows: all patients are referred to PET/CT, and the state of a final diagnosis represents the final state for the patient. Patient-level outcomes were true positive (TP), false positive (FP) or negative. By definition, in a cohort of men with biochemical recurrence, any negative scans are false negative; in the majority of these cases, the scan is negative due to subclinical disease; in rare cases, this is the result of PC with low or absent PSMA expression [21]. Further details are as outlined in the supplementary materials.

The resultant Markov chains were visualised by implementing them in MATLAB (MathWorks, Vers 5.3.1). Mixing times, the time until the Markov Chain reaches its steady state, were obtained from MATLAB. Differences between rates of findings were interrogated by means of the chi-square test, with *p* < 0.05 being considered statistically significant. The uncertainty in each state transition was propagated through the model using beta distributions based on the observed numerator and denominator information with 95% credible confidence intervals calculated using the beta distribution as previously described [22]. This was implemented in R using R Studio [23].

### Data inputs

The transition matrix was implemented using the transition probabilities obtained from the clinical data. Rates of positive and negative scans, rates of uncertain findings and the rate at which follow-up or additional imaging was performed were evaluated by scrutiny of patient notes to clinical follow-up. Scrutiny of all patients’ clinical records was performed for validation of scan results to a composite reference standard of truth (CSOT). In brief, confirmation of positive findings was defined as those where local therapy in the absence of systemic therapy caused a fall in PSA, where confirmative histology was available, where correlative imaging (CT, MRI, bone scan) was available or where imaging response to therapy could be ascertained [24]. The details are as outlined in Supplementary Figure S2. For those patients where a CSOT is available, the PPV could be defined from the number of confirmed TP and FP scans.

However, noting that in early biochemical recurrence a number of patients remain castration sensitive, many patients are referred to systemic androgen deprivation therapy without further imaging or biopsy, and as such no CSOT can be available for every patient. To minimise any potential bias, where PPV is dependent upon the prevalence [25], we use literature-derived values obtained by narrative review to capture a more accurate and generalisable picture of tracer performance. In a hierarchy of evidence, meta-analyses were preferred where available and their quality assessed according to the Preferred Reporting Items Of Systematic Reviews and Meta-Analyses (PRISMA) statement [26]. Where meta-analyses were not available, a random-effects meta-analytic model was used to obtain a synthesised value for the PPV, and compared with the estimated PPV obtained by composite follow-up. The quality of the studies synthesised was assessed by the QUADAS-2 tool [27].

### Evaluating radioligand performance

From the network as described above, the final probability of being assigned either a TP or a FP was calculated, from which the overall PPV can be obtained. The lack of true negatives at a patient-based level precluded specificity or NPV analysis. Diagnostic “harm” was defined as the rate of FP diagnoses, and diagnostic “benefit” was defined as the number of patients receiving TP diagnoses. A false discovery rate (FDR) could be defined as the rate of false diagnoses (FP and negative scans, where at a patient level all scans are formally false negative) as a proportion of total findings. From these data, analogous to the concept of “number needed to treat”, a “number needed to image” (NNI) could be calculated. NNI = 1/ARR, with ARR = absolute risk reduction, and is defined as ARR = CER – EER (control event rate CER – experimental event rate EER, where the event rate is defined as the rate of a TP). Likewise analogous to the “number needed to harm”, a “number needed to image to harm” (NNTITH) can be calculated, where the event rate is a false diagnosis (FP). Network properties were interrogated by evaluation of the eigenvalues of the transition matrices on the complex plane, which is a measure of the mixing time for each network (i.e. time taken until a steady state is reached).

### Economic analysis

Costs can be assigned to each imaging or diagnostic procedure. Local costs were costs obtained from the official Swiss medical reimbursement tariff system (tariff médical, TARMED). To aid international comparison, a selection of indicative prices were sought for the following indicative jurisdictions where both tracers are in routine use: Denmark, Germany, Israel, Australia and the UK. As an aid for international comparison, prices are presented in local currency and in OECD purchasing parity dollars (https://stats.oecd.org/Index.aspx?DataSetCode=SNA_TABLE4), which afford an improved international comparison of prices (https://www.oecd.org/health/health-systems/Health-Care-Prices-Brief-May-2020.pdf). An incremental cost per outcome (cost per TP finding) can be defined as *O*_*a*_*-O*_*b*_ where *O*_*a,b*_ represents the cost per outcome for tracers *a* and *b*. An incremental cost-effectiveness ratio (ICER) can be defined as *ICER* = (*C*_a_-*C*_b_)/(*E*_a_-*E*_b_), where *C*_a,b_ are the per-patient costs of tracers *a* and *b*, and *E*_a,b_ is the outcome effect (in this case, a TP diagnosis). The prices used are as outlined in supplementary Table S2. For cost efficacy decision-making, the willingness to pay threshold was taken to be the PET/CT scan cost in that country [28].

## Results

### *Clinical performance [*^*68*^*Ga]Ga-PSMA-11*

**Fig. 2:**
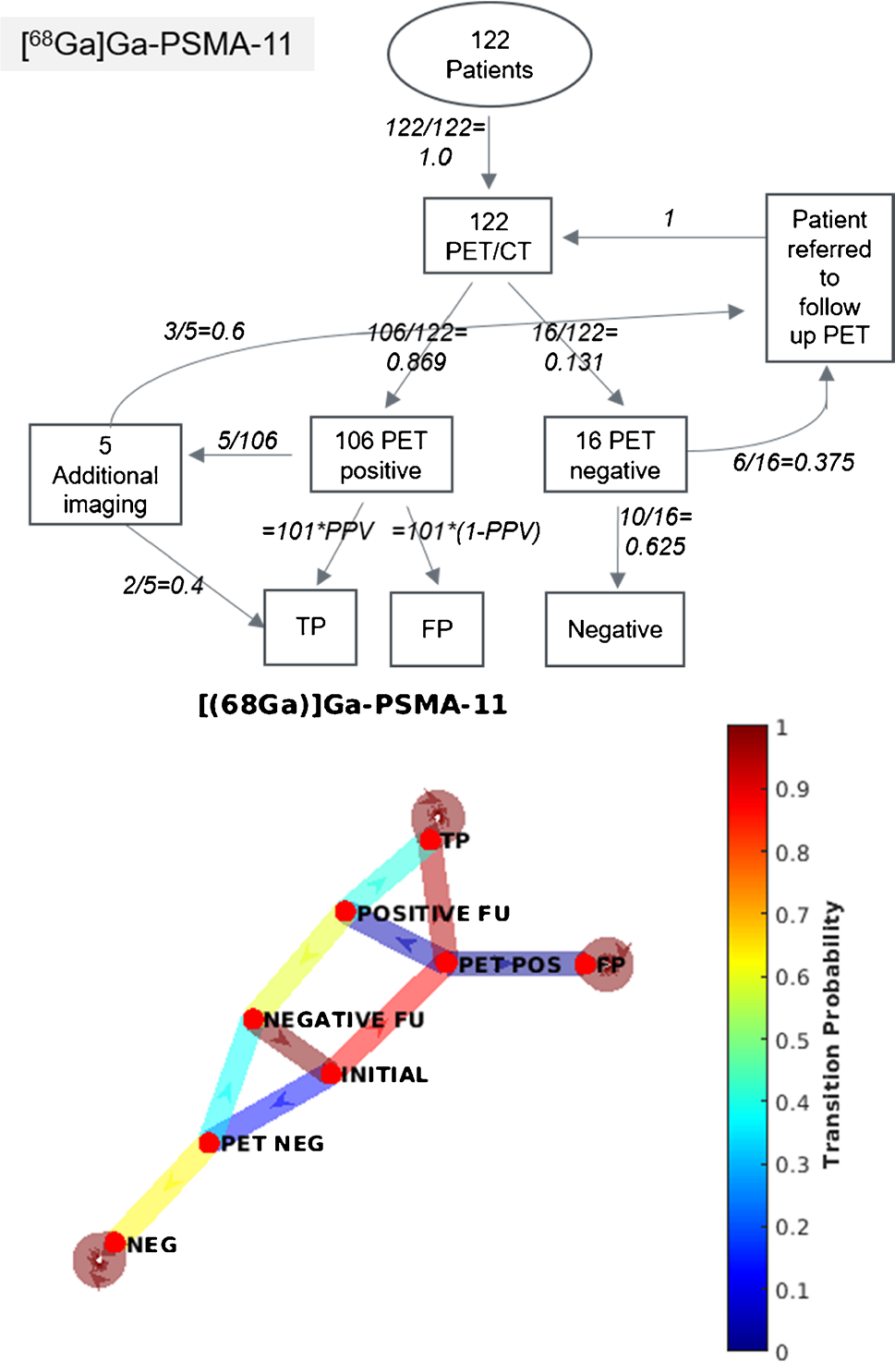
Markov transition chain for [^68^Ga]Ga-PSMA-11. Shown are the probabilities for the individual states as defined above and the transition probabilities in italics. Of the patients referred to further imaging, 3/5 were referred for follow-up PET/CT, 1 for histology (biopsy) and 1 for MRI; both cases were confirmed as TP. In the image below, the transition matrix is plotted as a heat-map, where the colour of the edge represents the individual transition probability and the node each state

*N* = 122 men underwent PET/CT with [^68^Ga]Ga-PSMA-11, of which 16 were PET-negative (PET positivity rate = 86.9%). Of the *n* = 16 men with negative PSMA PET/CT, six additional follow-up PET/CT were performed. Of the *n* =106 men with a positive PET, ten had uncertain positive findings (8.2% rate of uncertain findings). Of these uncertain findings, five patients proceeded to additional follow-up (1 PET/CT guided biopsy, 1 MRI and 3 were referred for follow-up PSMA PET/CT). Both the PET/CT-guided biopsy and the MRI confirmed PC lesions. For the 106 men with positive findings, 46 men had a composite standard of truth (CSOT) at follow-up, with 43 TP and 3 FP, yielding a PPV for these patients of 0.93 (95% confidence intervals (CI) 0.72–0.99). Cases where FP lesions were found are outlined in supplementary Table S3. The Markov transition chain is given in Fig. 2 and the probability matrix in supplementary materials. The overall probabilities of each end state and the final PPV are in Table 1.

**Table 1:** Final probabilities for a true positive (TP), false positive (FP) and a negative finding. The model-derived overall positive predictive value (PPV). Lower (LCI) and upper (UCI) 95% confidence intervals are given. *P* values are given in the final column

	[^68^Ga]Ga-PSMA-11			[^18^F]PSMA-1007			***P***
	Value	95% LCI	95% UCI	Value	95% LCI	95% UCI	
True positive	0.90	0.70	0.98	0.81	0.66	0.91	0.19
False positive	0.01	0.00	0.01	0.13	0.00	0.20	< 0.0001
Negative	0.09	0.02	0.97	0.06	0.01	0.94	0.91
PPV	0.99	1.00	0.99	0.86	1.00	0.82	< 0.0001

### *Clinical performance [*^*18*^*F]PSMA-1007*

**Fig. 3:**
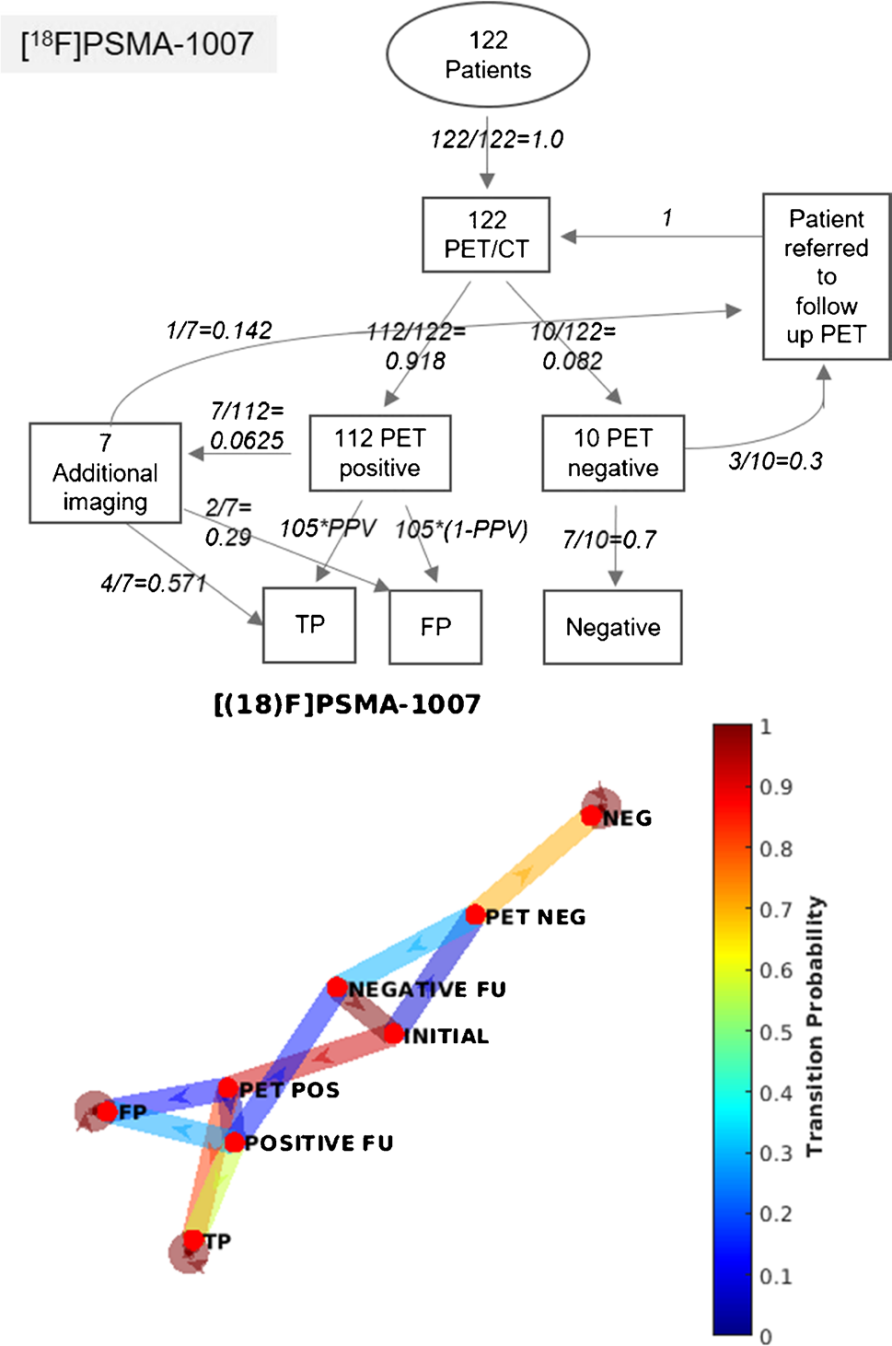
Markov transition chain for [^18^F]PSMA-1007. Shown are the probabilities for the individual states as defined above and the transition probabilities in italics. Of the patients referred to further imaging, 1/7 was referred for follow-up PET/CT, 2 for histology (biopsy) and 1 for CT, confirming and 3 for MRI, two of which confirmed a FP and 1 a TP. In the image below, the transition matrix is plotted as a heat-map, where the colour of the edge represents the individual transition probability and the node each state

*N* = 122 men underwent PET/CT with [^18^F]PSMA-1007, of which 10 were PET-negative (PET positivity rate = 91.8%, and was marginally higher than [^68^Ga]Ga-PSMA-11 without statistical significance, *p* = 0.68). Of the *N* = 10 men with negative PSMA PET/CT, three were redirected to follow-up PSMA PET/CT. Of the *N* = 112 men with a positive PET, 21 had uncertain findings (17.2%) which was significantly higher than for [^68^Ga]Ga-PSMA-11 (*p* = 0.02). Of these uncertain findings, seven patients underwent further imaging (one additional PET/CT, two PET/CT-guided biopsies, one CT and three MRI). Of the six patients who were not referred for a follow-up PET/CT, four had PC lesions confirmed and two identified were false positives. Of the 112 patients with positive scans, CSOT was available for 54 patients, with 45 TP and nine FP, yielding a PPV of 0.83 (95% CI 0.65-0.94). Cases where FP lesions were found are outlined in supplementary Table S3. The Markov transition chain is given in Fig. 3 and the transition matrix in supplementary materials. The overall probabilities of each end state and the final PPV are in Table 1.

### Literature-derived diagnostic accuracy data

For [^68^Ga]Ga-PSMA-11, the recent meta-analysis of Hope et al. was available and was analysed for quality using the PRISMA statement [29]. Hope et al. calculated a sensitivity of 0.74 (95% CI 0.51–0.89) and specificity 0.96 (95% CI 0.85–0.99) using nodal pathology as gold standard [30]. The PPV was found to be 0.99 (95% CI 0.96–1.00), with no significant difference compared to the PPV observed at follow-up in our cohort (*p* = 0.84).

For [^18^F]PSMA-1007, no such meta-analytic data for diagnostic accuracy is reported. A systematic literature review performed in July 2021 by the first two authors for the key search terms “PSMA-1007”, “diagnostic accuracy” and “performance” using the PubMed Central archive. This search revealed only three studies reporting data regarding the diagnostic performance of this tracer. Rahbar et al. and Giesel et al. report a 95% rate of positive scans, but do not provide confirmatory follow-up [31, 32]. Sprute et al. report data regarding diagnostic accuracy for nodal staging only in a mixed cohort of primary and recurrent patients, with a PPV of 0.913 [33]. Witkowska-Patena et al. report data for individuals with < 2.0 ng/ml, with a PPV of 0.667. These PPV data were combined using a random-effects model, with the forest-plot shown in supplementary Figure S3, where the synthesised PPV was 0.87 (95% CI 0.82–0.91) and is consistent with the PPV observed at follow-up in our cohort (no significant difference, *p* = 0.54). QUADAS-2 assessment revealed low risk of bias for both studies (Witkowska-Patena and Sprute et al.), with unclear risk of applicability (data presented in supplementary materials).

### Markov chain analysis

In our study, the overall PPV for [^68^Ga]Ga-PSMA-11 was 0.99 (95% CI 0.99–1.0) and was significantly higher (*p* < 0.0001) than the value for [^18^F]PSMA-1007 0.86 (95% CI 0.82–1.00). The FDR was lower for [^68^Ga]Ga-PSMA-11 (0.097 vs. 0.19, *p* < 0.005). The results are shown in Table 1. The probability for a patient to receive a TP diagnosis (the desired outcome) is 0.90 (95% CI 0.70–0.98) for [^68^Ga]Ga-PSMA-11 and 0.81 (95% CI 0.66–0.91) for [^18^F]PSMA-1007 (*p* = 0.20). The NNI was therefore 10.58, suggesting that for roughly every tenth patient imaged with [^68^Ga]Ga-PSMA-11 instead of [^18^F]PSMA-1007, one extra true positive scan would be observed. The lower PPV and higher rate of FP scans (0.13, 95% CI 0.00–0.20 for [^18^F]PSMA-1007 vs. 0.01, 95% CI 0.00–0.01 for [^68^Ga]Ga-PSMA-11, *p* < 0.0001) means that the number needed to image to harm (NNTITH) is − 8.08, suggesting that for every eighth patient imaged with [^18^F]PSMA-1007, potential diagnostic harm may occur as a result of a false positive finding. No significant difference was observed in the likelihood of a negative finding (0.09 [^68^Ga]Ga-PSMA-11 vs. 0.06 [^18^F]PSMA-1007, *p* = 0.91), where the wide confidence intervals seen in Table 1 are as a result of the low numbers of patients with a final outcome of a negative scan for both tracers. Mixing times were t_Mix_= 0.9829 and 1.3133 for [^18^F]PSMA-1007 and [^68^Ga]Ga-PSMA-11, respectively.

### Economic performance

Costs associated with each examination and associated follow-up imaging are outlined in Table 2. For the jurisdictions of interest, publically available cost data was available for Denmark (Danish Health Authority, Casemix360 (sundhedsdata.dk)), Israel (Ministry of Health, https://www.health.gov.il/English/Topics/finance/Pages/default.aspx) and Australia (Department of Health Medical Costs Finder | Australian Government Department of Health, with PSMA-PET costs as previously published, Gordon et al. [18]). For Germany, PSMA-PET/CT is not covered by statutory insurance; the private costs vary from clinic to clinic and are not publically available. For the UK, the cost details for PET/CT imaging is not included in publically available NHS National Tariff framework (https://www.england.nhs.uk/pay-syst/national-tariff).

**Table 2:** Incremental costs ([^68^Ga]Ga-PSMA-11 versus [^18^F]PSMA-1007) per patient, per outcome and the incremental cost-effectiveness ratio (ICER) for the four regions analysed, with prices in purchasing parity dollars ($)

Region	Incremental cost per patient	Incremental cost per TP	ICER
Switzerland	$109	− $243	$1153
Israel	$67	− $175	$707
Denmark	$485	$348	$5135
Australia	$26	− $64	$280

Using price data for Switzerland, the mean cost per patient, including all additional examinations, is lower for [^18^F]PSMA-1007 at $3212 vs. $3337 for [^68^Ga]Ga-PSMA-11. At an outcome level, the cost per TP favours [^68^Ga]Ga-PSMA-11 at $3697 per TP vs. $3975 per TP for [^18^F]PSMA-1007. The incremental cost per outcome favours [^68^Ga]Ga-PSMA-11 at $109, meaning that each TP finding would cost only an additional $109. The incremental cost efficacy ratio was strongly positive at $1153 for [^68^Ga]Ga-PSMA-11 compared to [^18^F]PSMA-1007, suggesting that while higher overall costs are associated with the use of [^68^Ga]Ga-PSMA-11 in Switzerland, these are less than 50% of the cost of a PET/CT and fall under the willingness to pay threshold (cost of a PSMA PET/CT in Switzerland is $2737.88) [28]. Cost efficacy per TP and the ICER favoured [^68^Ga]Ga-PSMA-11 for Switzerland, Israel and Australia but not Denmark, where the lower radiopharmaceutical costs for [^18^F]PSMA-1007 favoured this tracer, and where the ICER ($5135) was beyond the willingness to pay threshold (cost of a PSMA-PET/CT in Denmark $1387.90 to $1786.11) (Table 2 and supplementary Table S2).

## Discussion

In this study we present a Markov chain decision analysis which compares the relative performances of two PSMA radioligands under clinical conditions. Consistent with published retrospective analyses [31, 34] and meta-analysis [7], we find that the PET-positivity rate for [^18^F]PSMA-1007 is marginally, but non-significantly higher when compared to [^68^Ga]Ga-PSMA-11 (our data: 91.8% vs. 86.9%, *p* = 0.68). However, we find differences between the two tracers in terms of clinical performance and cost efficacy.

Whereas a number of studies reporting the diagnostic performance for PSMA-radioligands report findings on a binary positive/negative scale and without external validation of findings, this dialectic does not reflect clinical reality, where diagnostic uncertainty can and does occur. Furthermore, the patient’s journey does not end with a “positive” or “negative” scan result; any imaging findings inform clinical decision-making and influence subsequent treatment. Although a number of studies investigate the influence of PSMA-PET/CT on treatment decision-making [35], few adequately take into consideration the process of this complex and multi-disciplinary clinical decision-making process. Furthermore, no comparative data for the various PSMA radioligands has hitherto been reported in this regard [36]. Reports of crude detection rate or frequency of uncertain findings rarely consider statistical uncertainty in the observed data, or how this propagates, which our Bayesian model takes into account [37].

The choice of radioligand may have both clinical and healthcare economic implications. For example, a number of reports describe increased non-specific radioligand uptake with [^18^F]PSMA-1007 compared to [^68^Ga]Ga-PSMA-11 [8]. Indeed, Kuten et al. find that 13/15 (87%) patients undergoing [^18^F]PSMA-1007 PET/CT (albeit in primary PC) had equivocal bone lesions, of which a sizeable minority (11%) were TP findings [38]. These studies lend support to our observation that the rate of indeterminate findings was significantly higher for [^18^F]PSMA-1007 compared to [^68^Ga]Ga-PSMA-11 (17.2% vs. 8.2%, *p* = 0.02). This translated into a higher rate of additional examinations to clarify uncertain findings, which more often confirmed false positives. Confirmatory follow-up data to a CSOT was available for roughly half of the patients, allowing a direct estimate of the diagnostic accuracy. By comparing the model-derived probabilities for a TP and a FP, the overall patient-level PPV can be calculated. We find that the PPV for [^68^Ga]Ga-PSMA-11 was significantly higher than for [^18^F]PSMA-1007 (0.99 vs. 0.86, *p* < 0.001), which corresponded almost perfectly with literature/meta-analysis-derived values for the PPV used in our model (0.99 vs. 0.87), and is taken to be suggestive that there was no significant bias in patient selection in our study or methodology. The lower PPV for [^18^F]PSMA-1007 might be explained by the higher rate of non-specific uptake, for which further studies are required. As a result, the FDR was lower (0.09 vs. 0.19, *p* < 0.0005) and the overall probability for a patient to receive a TP was slightly higher for [^68^Ga]Ga-PSMA-11 (0.90 vs. 0.81, *p* = 0.19).

Using the concept of the number needed to treat, we provide an assessment of the effectiveness of PSMA-PET/CT as a healthcare intervention. We calculate a number needed to image, finding that when imaging with [^68^Ga]Ga-PSMA-11 instead of [^18^F]PSMA-1007, every tenth patient will benefit through an additional TP (NNI = 10.58). A number needed to image to harm reveals that every eighth patient will suffer an end result of a false positive (NNTITH − 8.08) when scanned with [^18^F]PSMA-1007. Using clinical data, we are therefore able to quantify, for the first time, the potential clinical impact of choosing one PSMA-radioligand over another.

Through the integration of cost data, we are able to provide a direct cost efficacy comparison for these two radioligands. For the reasons outlined above, our cost efficacy analysis reveals higher overall incremental costs per patient for [^68^Ga]Ga-PSMA-11 (ranging from an additional $26 per patient in Australia to $109 in Switzerland and $485 in Denmark, Table 2). However, the average cost per patient does not constitute an analysis of the cost efficacy of a healthcare intervention; a cheaper but less effective examination would not be cost-effective. The cost per desired outcome (a TP finding) was lower for [^68^Ga]Ga-PSMA-11 when compared to [^18^F]PSMA-1007 in all jurisdictions analysed except Denmark. An estimated 365,000 men develop prostate cancer in the European Union annually (https://publications.jrc.ec.europa.eu/repository/handle/JRC101382) and an estimated 25,000 men develop rPC annually in the USA [39]. With these large potential numbers of patients in mind, the choice of radiopharmaceutical might have substantial cost implications for a healthcare system. Although our data suggest a slightly higher detection rate for [^18^F]PSMA-1007, we find that this translates neither into improved clinical performance nor higher cost efficacy [40].

One notable finding of this examination was the variable degree of opacity in the reporting of economic data. For two jurisdictions (the UK and Germany), no open-access cost data was available, meaning that a cost efficacy analysis for these two countries was not possible, whereas for Israel and Australia, these data are published on the respective health ministry websites. In the Swiss TARMED system, the amount billed for the radiopharmaceutical is equal irrespective of tracer, and any cost savings incurred by using a different radiopharmaceutical are passed on to neither patient nor insurer. Similarly, no evidence of variation in tracer-specific costs could be found for Israel and Australia. Interestingly, in Denmark, the examination cost for a [^18^F]PSMA-1007 PET/CT was lower, resulting in improved cost efficacy for this tracer for Denmark. We were unable to assess further the costs of radiopharmaceutical choice on system-level costs; for example, although generator costs may be higher, [^68^Ga]Ga-PSMA-11 is not under a patent, whereas production of [^18^F]PSMA-1007 incurs licence fees to the patent holder.

To facilitate future analyses in other jurisdictions or for other tracers, we provide a model which is straightforward to implement in Microsoft Excel using local data. In addition to the above, periods of ^68^Ga non-availability due to generator shortages or low generator yields, and resultant scan cancellations or re-bookings must also be considered when choosing which of these two radioligands to routinely implement, particularly where scanner time is an increasingly limited commodity. Given the improved clinical performance of [^68^Ga]Ga-PSMA-11 revealed by our analysis, cyclotron produced ^68^Ga [41] or ^18^F-labelled PSMA-11 [42] may be areas where the cost efficacy of PSMA-PET/CT can be further improved.

Although not a study-specific weakness, we draw attention to the fact that the PPV for to [^68^Ga]Ga-PSMA-11 is confirmed by a large volume of prospective data and meta-analysis [30]. This was not the case for [^18^F]PSMA-1007, where only two studies reporting data for PPV were identified, and several prospective studies are underway. In mitigation all available data for [^18^F]PSMA-1007 was systematically reviewed and combined in a meta-analytic model and study quality was formally assessed. In light of the widespread adoption of this tracer, further studies are required to confirm the lower PPV for [^18^F]PSMA-1007. In two countries (UK and Germany), a cost efficacy analysis was hampered by the lack of publically available health economic data for PSMA-PET scans. Greater transparency in the reporting of these data would be welcome to facilitate future health-economic analyses for nuclear medicine imaging.

These retrospective data require further studies to be confirmatory, although we note that strict consecutive inclusion and non-overlapping dates in radioligand availability limit selection bias. Our study is restricted to a single centre analysis, and further multi-centre and ideally international studies should be performed. Finally, our analysis is limited to a pairwise analysis of [^18^F]PSMA-1007 and [^68^Ga]Ga-PSMA-11. We urge further similar studies from centres with experience of other radioligands, where our model is generalisable and implementable with locally obtained values for other tracers.

## Conclusion

We present a generalisable Markov decision model analysis informed by clinical data obtained from a retrospective analysis of a large single-centre cohort of patients to compare the clinical performance and cost efficacy of the two PMSA-radioligands [^68^Ga]Ga-PSMA-11 and [^18^F]PSMA-1007. In keeping with previous studies [7], we find a slightly higher PET-positivity rate for [^18^F]PSMA-1007, but find significantly greater rates of uncertain findings and false positives for this tracer when compared to [^68^Ga]Ga-PSMA-11. The lower PPV for [^18^F]PSMA-1007 observed in our cohort was in-keeping with literature-derived values and a meta-analytic synthesis of existing data. The higher frequency of false positives, additional imaging or additional intervention to clarify a higher rate of indeterminate findings are indicative of a less favourable clinical performance for [^18^F]PSMA-1007 compared to [^68^Ga]Ga-PSMA-11. We find significant differences in terms of clinical performance and cost efficacy for these two radioligands, and the choice of which to implement should be informed by these as well as local conditions, with variation in cost efficacy across the jurisdictions analysed herein.

## Supplementary Information

Below is the link to the electronic supplementary material.

Supplementary file1 (DOCX 260 KB)

## Declarations

The study was performed in accordance with the Helsinki declaration. The study was approved by the institutional review board (IRB) KEK ID 2020-01162.
