# Supplementary material for: Comparing the clinical performance and cost efficacy of [68Ga]Ga-PSMA-11 and [18F]PSMA-1007 in the diagnosis of recurrent prostate cancer: a Markov chain decision analysis
Source: Eur J Nucl Med Mol Imaging. 2021 Nov 13;49(12):4252–61. doi: 10.1007/s00259-021-05620-9 (PMC9525363; doi:10.1007/s00259-021-05620-9)
Supplement: Supplementary file 1 — Supplementary file1 (DOCX 260 KB) [file 259_2021_5620_MOESM1_ESM.docx]

Supplementary Materials

**Markov Chain**

As shown by the network of states in Supplementary Figure S1, patients following a scan first receive either a positive or negative PET result. Depending on clinical context, a patient with a negative scan can undergo either further therapy such as hormone deprivation, or, noting that the detection rate is dependent upon PSA value ^1,2^, a repeat PET/CT can be recommended, and the patient returns to the beginning of the flow-chart. The likelihood of a positive finding being either TP or FP depends the tracer’s PPV, where PPV = TP/(TP + FP). In some cases, PSMA-avid lesions can be reported as uncertain or equivocal, and a rate of “uncertain finding” can be defined. These may then be clarified either by repeating the PET/CT after a certain period, or by performing complementary imaging or a biopsy (“rate of additional imaging”).

The Markov-chain formalism models a patient’s transition through these various states as outlined above. Each probability can be represented as a transformation matrix, where the Probability P to move from state i to j is Pr(j|i) = Pi,j, and the total probability to move from a state i to all other states is 1. The Markov property determines that the likelihood of the next trajectory is dependent on the previous, for example, the likelihood of being referred to follow- up PET/CT upon a negative PET is dependent on the likelihood of a negative PET. The various states are represented by a stochastic probability matrix P=(Pij), and the probabilities are given by the product of this matrix and a stationary probability vector π(n); $\pi_{0}^{T}\boldsymbol{P}=\pi_{1}^{T}$ . We assume that the probability of each state is fully observable and that the system is autonomous (i.e. probabilities of each state are dependent on the tracer properties). The transition probabilities were informed by clinical data, and where appropriate, literature derived values were obtained.

Initial modelling assumptions were as follows: all patients are referred to PET/CT and the state of a final diagnosis represents the final state for the patient. Patient-level outcomes were true positive (TP), false positive (FP) or negative. By definition in a cohort of men with biochemical recurrence any negative scans are false negative; in the majority of these cases the scan is negative due to subclinical disease, in rare cases this is the result of PC with low or absent PSMA-expression.

**Imaging routines**

Imaging was performed as per institutional standard at 90min p.i. with a mean activity of 243 (range 133-322) MBq for [68Ga]Ga-PSMA-11 or a mean activity of 246 (range 207-283) MBq at 2h p.i. for [18F]PSMA-1007 in accordance with our clinical routine. All patients underwent imaging at the University Clinic for Nuclear Medicine, Bern. Image acquisition and reconstruction parameters were as previously published 1,3.

Scans with at least one PSMA-avid lesion suspicious for rPC were recorded as “positive” at a patient-based level and those with no PSMA-avid lesions suspicious for rPC were recorded as “negative” at a patient-based level (= PET-positivity rate).

**Supplementary Tables and Figures**

**eTable S1.** Patient characteristics and previous therapies/therapeutic combinations (RPE = radical prostatectomy; RT = radiotherapy; ADT = androgen deprivation therapy)

| **Parameter** | **[^68^Ga]Ga-PSMA-11** | **[^18^F]PSMA-1007** | **p** |
| --- | --- | --- | --- |
| Activity [MBq] *mean (range)* | 243 (131-322) | 246 (207-283) | - |
| Age [yr] *mean (range)* | 71 (52-85) | 72 (54-87) | 0.23 |
| PSA [ng/ml] *median (range)* | 2.75 (0.2-4513) | 2.23 (0.12-518) | 0.38 |
| GS median *(range)* | 7 (5-10) | 7 (5-10) | 0.71 |
| *Previous therapies:*  RPE  RT  ADT  RT+ADT  RPE+ADT  RPE+RT  ADT+Chemotherapy+RT  Brachytherapy | 92  5  6  6  1  3  0  2 | 90  5  16  3  2  1  1  1 | - |
| T *median* *(range)* | 3 (1-4) | 3 (1-4) | 0.38 |
| N *median (rang*e) | 0 (0-3) | 0 (0-1) | 0.39 |
| M *median (range)* | 0 (0-1) | 0, 0-1 | 0.91 |

**eTable S2.** Cost inputs, where MRI is of the pelvis without contrast medium, Biopsy is a CT-guided bone biopsy and ceCT is a contrast enhanced scan of the thorax. Prices in Swiss Francs (CHF) at current official tariff (TARMED) prices and in OECD purchasing parity power US Dollars ($).

| **Cost Inputs** | **Switzerland** | | **Israel** | | **Denmark** | | **Australia** | |
| --- | --- | --- | --- | --- | --- | --- | --- | --- |
|  | **CHF** | **PPP USD** | **NIS** | **PPP USD** | **DKR** | **PPP USD** | **AUD** | **PPP USD** |
| ^18^F-PSMA-1007 PET/CT | 3050.00 | 2737.88 | 6642 | 1801.95 | 11783 | 1786.11 | 1000 | 680.2721 |
| ^68^Ga-PSMA-11 PET/CT | 3050.00 | 2737.88 | 6642 | 1801.95 | 9156 | 1387.90 | 1000 | 680.2721 |
| MRI pelvis w/o contrast | 2319.00 | 2081.69 | 2034 | 551.82 | 2319 | 351.52 | 627.2 | 426.6667 |
| CT Biopsy | 4781.00 | 4291.74 | 5609 | 1521.70 | 4781 | 724.72 | 275 | 187.0748 |
| ceCT | 2007.00 | 1801.62 | 700 | 189.91 | 2007 | 304.23 | 588 | 400 |

**eTable S3.** Cases of false positive studies with details of additional examinations performed to confirm these.

| **^18^F-PSMA-1007**: | |  |  |  |  |  |  |  |  |  |  |
| --- | --- | --- | --- | --- | --- | --- | --- | --- | --- | --- | --- |
| 2 FP underwent additional F-18-PSMA-1007 PET without any pathology | | | | | | |  |  |  |  |  |
| 3 FP underwent additional MRI, two of the pelvis (bone lesions) one of the abdomen without confirmation of the unclear PSMA-avid lesions. | | | | | | | | | | | |
| 4 FP had rising PSA after targeted RT and were therefore counted as FP. | | | | | | |  |  |  |  |  |
|  |  |  |  |  |  |  |  |  |  |  |  |
| **^68^Ga-PSMA-11:** | |  |  |  |  |  |  |  |  |  |  |
| 1 FP underwent additional ^18^F-PSMA-1007 without any pathology | | | | | | |  |  |  |  |  |
| 1 had a negative histology of a positive PSMA-lesion (mediastinal LN) | | | | | | |  |  |  |  |  |
| 1 underwent additionally MRI of the pelvis without confirmation of a reported PSMA-avid local recurrence. | | | | | | | | | |  |  |

**eFig S1.** Schematic of the Markov decision process. The patient first receives a PET/CT, which generates either a positive or negative finding. PET negative findings can result in re-referral to PET/CT (negative follow-up rate) or where PET positive findings can generate additional examinations (positive follow-up rate). These probabilities were informed by real-life clinical data sampled from our retrospective cohorts. The probability of true positive (TP), false positive (FP), true negative (TN) and negative scans were informed by reported data for the diagnostic performance for each tracer.


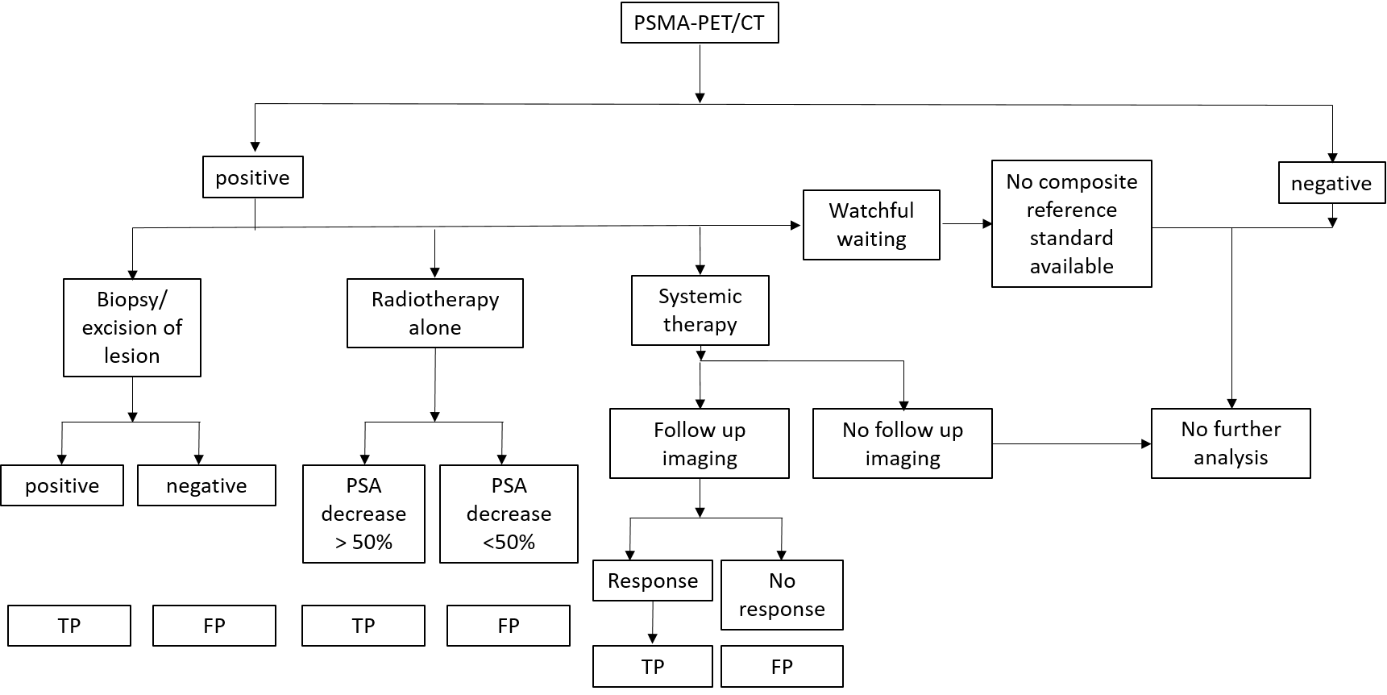


**eFig S2.** Details of the reference standard to confirm or refute PET findings. A composite standard of truth included histology findings, response to therapy and comparative imaging findings.

**
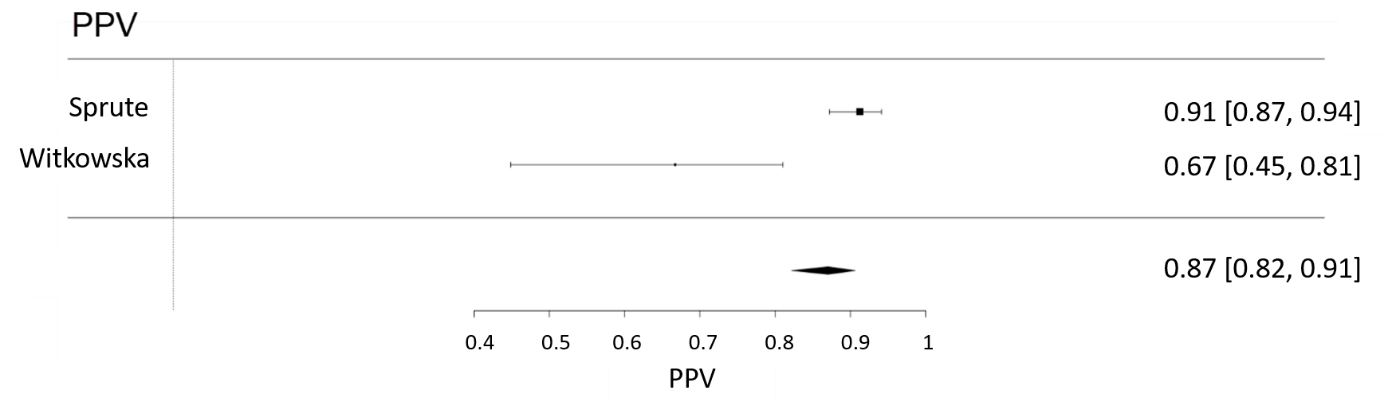
**

**eFig S3.** Mini meta-analysis for the two available publications reporting PPV data for [^18^F]PSMA-1007, Sprute et al.^4^ and Witkowska-Patena et al.^5^, yielding an overall PPV of 0.87 (95% CI 0.82-0.91).

**References**

1. Alberts I, Prenosil G, Sachpekidis C, et al. Digital versus analogue PET in [(68)Ga]Ga-PSMA-11 PET/CT for recurrent prostate cancer: a matched-pair comparison. *Eur J Nucl Med Mol Imaging.* 2020;47(3):614-623.

2. Afshar-Oromieh A, Holland-Letz T, Giesel FL, et al. Diagnostic performance of 68Ga-PSMA-11 (HBED-CC) PET/CT in patients with recurrent prostate cancer: evaluation in 1007 patients. *European journal of nuclear medicine and molecular imaging.* 2017;44(8):1258-1268.

3. Alberts I, Sachpekidis C, Dijkstra L, et al. The role of additional late PSMA-ligand PET/CT in the differentiation between lymph node metastases and ganglia. *Eur J Nucl Med Mol Imaging.* 2020;47(3):642-651.

4. Sprute K, Kramer V, Koerber S, et al. Diagnostic Accuracy of 18 F-PSMA-1007 PET/CT Imaging for Lymph Node Staging of Prostate Carcinoma in Primary and Biochemical Recurrence *Journal of Nuclear Medicine.* 2020:jnumed.120.246363.

5. Witkowska-Patena E, Giżewska A, Dziuk M, Miśko J, Budzyńska A, Walęcka-Mazur A. Diagnostic performance of 18F-PSMA-1007 PET/CT in biochemically relapsed patients with prostate cancer with PSA levels ≤ 2.0 ng/ml. *Prostate Cancer and Prostatic Diseases.* 2020;23(2):343-348.
